# Discriminative value of pulse wave velocity for arterial stiffness and cardiac injury in prediabetic patients

**DOI:** 10.1590/1677-5449.202300762

**Published:** 2023-12-11

**Authors:** Stella Maris Firmino, Cássia da Luz Goulart, João Paulo Gregorio, Klaus Werner Wende, Fernanda Yuri Yuamoto, Lana Kummer, Emílio Martins Curcelli, Alessandro Domingues Heubel, Erika Zavaglia Kabbach, Polliana Batista Santos, Audrey Borghi-Silva, Renata Gonçalves Mendes, Ângela Mérice de Oliveira Leal, Meliza Goi Roscani

**Affiliations:** 1 Universidade Federal de São Carlos – UFSCar, São Carlos, SP, Brasil.

**Keywords:** **:** arterial stiffness, carotid-femoral pulse wave velocity, prediabetics, risk factors, rigidez arterial, velocidade da onda de pulso carotídeo-femoral, pré-diabéticos, fatores de risco

## Abstract

**Background:**

Prediabetes (PD) is defined as impaired fasting glucose and/or impaired glucose tolerance (IGT) and may be associated with high risk of cardiovascular injury. It is recommended that PD patients be screened for signs of arterial stiffness and cardiovascular injury to reinforce therapeutic strategies.

**Objectives:**

To identify pulse wave velocity values discriminative for arterial stiffness and cardiovascular injury in PD patients.

**Methods:**

A cross-sectional study was conducted with PD (N=43) and normoglycemic (N=37) patients who underwent clinical evaluation, arterial stiffness assessment by carotid-femoral pulse wave velocity (cfPWV) using SphygmoCor, laboratory blood analysis, investigation of morphological and functional cardiac variables by transthoracic echocardiogram, and assessment of carotid intima-media-thickness (CIMT) by carotid ultrasonography. A statistical analysis was performed using SPSS software and values of p<0.05 were considered significant.

**Results:**

A cfPWV cut-off value of 6.9 m/s was identified for IGT (Sensitivity [SE]: 74% and Specificity [SP]: 51%). Comparison of general data and risk factors between subsets with values above and below this cutoff value revealed higher rates of fasting glucose (p=0.02), obesity (p=0.03), dyslipidemia (p=0.004), early signs of left ventricle (p=0.017) and right ventricle (p=0.03) impaired diastolic function, and elevated CIMT in subjects with cfPWV ≥ 6.9m/s (p=0.04).

**Conclusions:**

In PD patients, a cfPWV cutoff of 6.9 m/s was considered a discriminative value for arterial stiffness. These findings highlight the value of early investigation of cardiovascular injury and aggressive therapy strategies with good control of risk factors in PD.

## Resumo

**Contexto:**

O pré-diabetes (PD) é definido como glicemia de jejum alterada e/ou tolerância à glicose alterada (TGA) e pode estar associado a alto risco de lesão cardiovascular. Recomenda-se discriminar quais pacientes com PD podem apresentar sinais de rigidez arterial e lesão cardiovascular para reforçar as estratégias terapêuticas.

**Objetivos:**

Identificar os valores discriminativos da velocidade de onda de pulso determinantes de rigidez arterial e lesão cardiovascular em pacientes com PD.

**Métodos:**

Estudo transversal em pacientes com PD (N=43) e normoglicêmicos (N=37) submetidos a avaliação clínica, avaliação da rigidez arterial pela velocidade da onda de pulso carótido-femoral (cfPWV) utilizando SphygmoCor, análise laboratorial de sangue, investigação de alterações morfológicas e variáveis cardíacas funcionais por ecocardiograma transtorácico e avaliação da espessura íntima-média carotídea (EIMC) pela ultrassonografia da carótida. A análise estatística foi realizada no *software* SPSS, e valores de p<0,05 foram considerados significativos.

**Resultados:**

Foi identificado um valor de corte cfPWV de 6,9 m/s para TGA (sensibilidade 74% e especificidade 51%). A comparação dos dados e fatores de risco entre valores acima e abaixo do valor de corte estabelecido revelou glicemia de jejum elevada (p=0,02), obesidade (p=0,03), dislipidemia (p=0,004), sinais precoces de função diastólica prejudicada do ventrículo esquerdo (p=0,017) e ventrículo direito (p=0,03) e maior EIMC em cfPWV ≥6,9m/s (p=0,04).

**Conclusões:**

Em pacientes com PD, o cfPWV de 6,9 m/s foi considerado um valor discriminativo de rigidez arterial. Esses achados reforçam que a investigação precoce da lesão cardiovascular e uma estratégia com terapia agressiva são valiosas no controle dos fatores de risco na PD.

## INTRODUCTION

Changes during periods of hyperglycemia and insulin resistance (IR), with increases in free fatty acids (FFA) and pro-inflammatory substances, may predispose to endothelial dysfunction. This occurs due to increases in vasoconstrictor agents and decreases in vasodilator substances, culminating in vessel smooth muscle dysfunction. This process leads to an increase in arterial stiffness (AS), which is known to be an important precursor of development of atherosclerosis.^1-3^ Increased mortality is related to a combination of pre-diabetes (PD) with peripheral IR, obesity, hypertriglyceridemia, decreased levels of high-density lipoprotein (HDL), and systemic arterial hypertension (SAH), among other associated factors. This demonstrates that components of the metabolic syndrome can be identified in PD patients before a definitive diagnosis of Diabetes mellitus 2 (DM2) is made.^4,5^

The role played by AS in the worsening of cardiovascular disease is related to increased rigidity of large arteries, and consequently, to increased ejection pulse velocity of the ventricle through the arteries, resulting in an early return of the reflected pressure wave. This can occur until there is an increase in pressure during left ventricle (LV) ejection. Early arrival of the reflected pulse during systole increases the overload on the LV and reduces perfusion of the coronary arteries during diastole.^6-8^

Carotid-femoral pulse wave velocity (cf-PWV) is a standard measure used to assess AS.^3,9-11^ Although several studies establish reference values for carotid-femoral PWV in different populations, these are not specific to assessment of PD or diabetic individuals. We only found studies in the literature reporting PWV reference values in European,^8^ Asian,^12,13^ Argentinian,^14^ and Brazilian^15^ papers.

Considering the above, it is worth evaluating more reliable measures that discriminate PWV values more accurately in PD individuals in order to deliver timely interventions, preventing onset or progression of CV diseases and providing more effective strategies to target treatment. Therefore, the aim of this study was to identify PWV values discriminative of AS and cardiovascular injury in PD patients.

## METHODS

This is a cross-sectional study approved by the UFSCar ethics committee (CAAE: 79225217.5.0000.5504 and Opinion Number: 3.218.078) and following the principles of the Helsinki Declaration. The study was conducted with a sample of 80 patients of both sexes, classified as PD (N=43) or normoglycemic (N=37), and aged between 37 and 59 years. All participants read and signed the free and informed consent form (FICF). This sample of patients has also been described elsewhere in a previous publication by our research group with different objectives and results.^16^ The study was carried out according to the recommendations of the STROBE statement.^17^

The study was publicized through social media (TV, radio, and internet) and individuals who were interested in participating were selected from October 2018 to January 2020. The participants were invited to attend the UFSCar Research Center and, after signature of the consent form, eligible patients underwent CV assessment and blood sample collection.

The inclusion criteria were as follows: individuals of both sexes, aged 35 to 59 years. At least two of the PD criteria listed below were required for a diagnosis of PD, to ensure greater reliability of selection of the study groups:

- Fasting Glucose (FG) from 100 to 125 mg/dL;
- Glucose tolerance test (GTT) from 140 to 199 mg/dL;
- Glycated Hemoglobin (HbA1c) from 5.7 to 6.4% (39 to 47 mmol/mol).

The values used were based on data provided by the American Diabetes Association (ADA).^18^

The exclusion criteria were as follows: patients with known or suspected cardiovascular disease; presence of stable angina; presence of ischemia in complementary tests, such as exercise stress test, myocardial scintigraphy, stress echocardiogram, tomography or hemodynamic study of coronary arteries; presence of symptoms or tests suggestive of heart failure, valve disease (stenosis or insufficiency) greater than a mild degree; individuals with a history of or exams suggestive (ultrasonography or hemodynamic study) of carotid artery disease; vascular surgery involving the carotid, femoral, or aortic arteries; presence of congenital heart disease; cognitive disorders that interfere with understanding of the experimental procedure; pregnancy; use of illicit drugs; use of any hypoglycemic or weight-reducing medications in the past 3 months; or presence of ADA^18^ criteria for diabetes diagnosis.

*Study design:* All study participants underwent clinical evaluation, blood sample collection, transthoracic echocardiogram, cf-PWV measurement, and carotid intima-media thickness (CIMT) measurement by carotid ultrasonography.

*Clinical evaluation:* Detailed anamnesis was performed by a cardiologist with assistance from the medical students from the research group, recording symptoms, personal history, and associated risk factors in accordance with current guidelines.^19^ Continuous use medications and dosages were also recorded. The general physical examination included collection of data on weight, height, body mass index, systemic blood pressure, and heart rate. The specific physical examination included detailed investigation of the respiratory and cardiovascular systems.

*Blood collection:* blood was drawn in the morning, with instructions to fast for 12 hours before collection, and used to perform routine laboratory tests indicated in the investigation of PD. Lipid and renal profiles (fasting blood glucose, HbA1c, total cholesterol, LDL-cholesterol, HDL-cholesterol, triglycerides, creatinine, urea and urine creatinine/albumin ratio, triglycerides Glucose (TyG) index and standard 2h 75g OGTT) were collected.

*cf-PWV*: was assessed by non-invasive measurement of the carotid-femoral PWV (cf-PWV), considered the gold standard by the American Heart Association.^7^ For this purpose, the adapted assessment protocol from the specific consensus was used.^20^ Pulse waves were obtained transcutaneously using SphygmoCor XCEL equipment (AtCor Medical®, Australia), placing transducers in the topography of the right carotid and right femoral artery. Measurement of cf-PWV was performed in the right hemibody, with the patient in the supine position, after a minimum of 10 minutes at rest. Two measurements were performed with no difference greater than 5% between them, taking the mean of the two measurements as the cf-PWV.^20^ The examination was performed by a single trained professional, with no interobserver variability.

*Echocardiography:* A Philips HD11 echocardiogram device, from the NUPECE laboratory, in the Second Unit of the Department of Medicine at UFSCar – São Carlos, was used. The standards and techniques recommended by the American Society of Echocardiography^21^ were employed. The Doppler-echocardiographic study was performed by a single examiner. Three consecutive measures were obtained of each variable. The echocardiogram was performed with a 2-5MHz sector transducer. Measurements in millimeters were taken for LV diastolic diameter (LVDD), LV systolic diameter (LVSD), posterior wall thickness (PP), left atrium diameter (LA), and aortic diameter (AO) using the equipment’s own cursor during the examination. *Flows:* transmitral diastolic flow was obtained with the transducer placed in the four-chamber apical position, enabling measurement of the E wave (E, cm/s) and the A wave (H, cm/s). The Tissue Doppler image was obtained in real time, in the four-chamber apical window. The volume sample was placed in the basal portion of the ventricular wall (mitral annulus), in the interventricular septum, and in the basal portion of the right ventricle wall (tricuspid annulus). Peak annular velocities were measured in early diastole (E´), atrial contraction (A´), and systole (s´). Left and right atrial volumes were obtained by the Simpson method, with planimetry in the 4-chamber apical window. In all patients, the LV ejection fraction (LVEF) was calculated using the Teicholz method and the Simpson method. Since the patients enrolled had no history of previous heart disease and did not show changes in regional contractility, the methods were considered similar. Moreover, since there were many similarities in the LVEF, the Teicholz method results were used for analyses due to its good accuracy and reproducibility in this population.

CIMT: Carotid ultrasonography examinations were performed by a single examiner using Philips HD11 equipment equipped with a 12-3 MHz linear ultrasonic transducer and an image recording system. Patients remained in the horizontal supine position with the head slightly tilted to the side contralateral to the carotid being studied. Intima-media thickness was obtained by the automated method, with intima-media thickness determination. Measurements were performed on the posterior wall of the left and right common carotids and the average of the two measurements was used for analyses. The transducer was placed on a 10mm segment of the posterior wall of the common carotid artery R and R in the distal third of the vessel. CIMT>1.5 was considered an atheroma plaque. Images were obtained and analyzed following the recommendations contained in the “Consensus Statement from the American Society of Echocardiography”^21^ and the “Mannhein Carotid Intima-Media Thickness Consensus (2004-2006).”^22^ Atherosclerosis plaques were also investigated and when present were classified according to echogenicity, using the Gray-Weale criteria.^23^

### Statistical analysis

The Shapiro-Wilk test was used to verify the normality of data distributions. Descriptive variables were listed as mean, standard deviation, and percentage (%). Comparisons between groups were performed with the chi-square and Fisher tests for categorical variables and the *t* test for variables with normal distribution and the Mann-Whitney test for variables with non-parametric distribution. Associations between variables were investigated using Pearson’s correlation test.

The Receiver Operating Characteristic (ROC) curve for the population was analyzed to identify the ideal descriptive threshold values considering age and the reference values for cf-PWV.^8^ Subsequently, significant areas under the ROC curve were identified at the cutoff points of the variables, with their respective sensitivity and specificity values. Sigma Plot Systat software, version 11.0,^24^ was used in the analysis. A significance level of p<0.05 was adopted for all statistical tests.

Regarding sample size, the number of patients needed to be assessed (n=71) was calculated assuming a 5% risk of α error, β of 95%, and an effect size of 0.4.

## RESULTS

**Figure 1:**
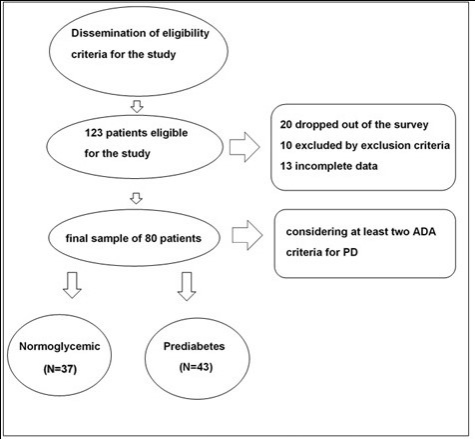
Sample criteria for inclusion and exclusion of patients.

There were 123 patients eligible for the study, with exclusion of 43 patients, as detailed in Figure 1. Twenty patients dropped out of the study, 10 patients were excluded according to the exclusion criteria and 13 patients were excluded for incomplete data. The final sample consisted of 80 patients, classified as PD (N=43) or normoglycemic (N=37).

Table 1 shows the baseline clinical characteristics of the individuals included in the study. We found predominance of women (60%) and of overweight patients. The most prevalent comorbidities were dyslipidemia (35%) and SAH (23.8%). Mean age was 48 years and the mean LV ejection fraction value was 68%.

**Table 1:** Characteristics of the study participants.

**Variable**	**Overall (N=80)**
	**N(%) or M(SD)**
Age, years	48.41 (10.63)
Sex, n (%)	
Female	48 (60.0)
Male	32 (40.0)
Body mass index, kg/m^2^	30.14 (5.91)
**Comorbidities**	
Dyslipidemia	28 (35.0)
Hypertension	19 (23.8)
Smoking	9 (11.2)
HbA1c, %	5.63 (0.39)
Glucose tolerance test, mg/dL	121 [88,143.25]
Pulse Wave Velocity, m/s	7.57 (1.69)
**Echocardiography assessment**	
LVDD, mm	48.12 (4.87)
E/E´ Mitral	7.09 (2.04)
E’ Mitral Tissue Doppler, cm/s	11.8(0.34)
E/A Tricuspid Inflow	1.40 (0.35)
LVEF, %	68 (11)
CIMT, mm	0.52 (0.07)

Continuous variables are presented as mean (M) ± standard deviation (SD) or median [interquartile range], whereas categorical variables are presented as absolute frequency (relative frequency). BMI: Body Mass Index; HbA1c: glycated hemoglobin; LVDD: Left Ventricle Diastolic Diameter; E: velocity of early diastolic mitral or tricuspid inflow; A: velocity of late diastolic mitral or tricuspid inflow; E’: mitral or tricuspid annular velocity of early diastole. LVEF: Left Ventricle Ejection Fraction; CIMT, Carotid Intima-Media Thickness. The characteristics of this sample are as described in a previous publication by our group.^15^

**Figure 2:**
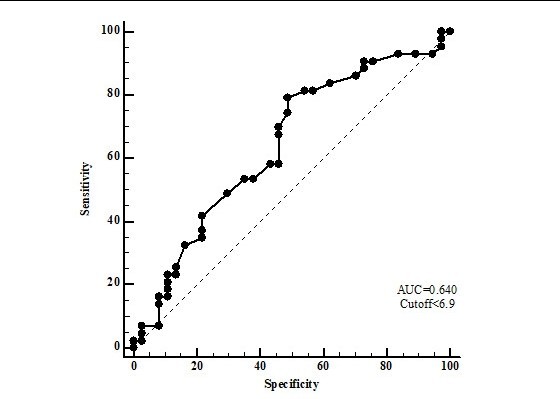
Receiver operating characteristic (ROC) curve for pulse wave velocity as an indicator of arterial stiffness for pre-diabetic patients. AUC: area under the curve.

An ROC curve was built to evaluate the potential of the cf-PWV variable to discriminate AS in PD patients (Figure 2) with an area under the curve (AUC) of 0.64 (CI: 0.52-0.74; p 0.02), a cf-PWW cutoff point of **≥** 6.9 m/s, sensitivity of 74%, specificity of 51%, and a Youden index of 0.97 for detection of AS and cardiovascular injury.

Table 2 shows a comparison dividing the subsets of patients with cf-PWV ≥ 6.9 and cf-PWV ≤ 6.9 m/s showing the variables age, BMI, glucose, HbA1c, arterial hypertension, dyslipidemia, and CV function. Individuals in the group with cf-PWV ≥ 6.9 m/s were older (p=0.001), had higher BMI (p=0.043) and higher glycemic fasting levels (p=0.022), had greater prevalence of dyslipidemia (p=0.004) and CV variables related to LV diastolic diameter, parameters of early diastolic dysfunction, assessed by E/A (p=0.017) and E’ Mitral Tissue Doppler (0.009), and had greater CIMT values (p=0.04).

**Table 2:** Comparison of general data and risk factors between patients with PWV values above and below the cutoff value established.

	**C-PWV<6.9 (n=28)**	**C-PWV≥6.9 (n=52)**	**p-value**
Age, years	43.3 ± 10.3	51.1 ± 9.9	**0.001**
BMI, kg/m^2^	28.3 ± 5.8	31.15 ± 5.8	**0.043**
Sex, N (%)			
Male	7(8.8)	25(31.3)	**0.044**
Female	21(26.3)	27(33.7)	
Glucose, mg/dL	102.5 ± 15.0	110.5 ± 14.6	**0.022**
HbA1c, %	5.5 ± 0.3	5.6 ± 0.4	0.159
Glucose Tolerance Test, mg/dL;	108.4 ± 29.6	126.8 ± 45.2	0.055
Dyslipidemia, n (%)	4(14.2)	24(46.1)	**0.004**
Triglycerides	133.1 ± 47.8	167.4 ± 81.4	**0.044**
HDL	48.3 ± 10.8	41.8 ± 9.8	**0.008**
**Echocardiography Assessment**			
LVDD, mm	46.5 ± 3.9	49.0 ± 5.1	**0.028**
LVSD, mm	30.6 ± 4.9	33.9 ± 6.0	**0.018**
E/A Mitral	1.4 ± 0.2	1.2 ± 0.3	**0.017**
E’ Mitral Tissue Doppler	13.1 ± 2.7	11.4 ± 2.9	**0.009**
E/A Tricuspid	1.5 ± 0.3	1.3 ± 0.3	**0.032**
CIMT	0.5 ± 0.06	0.5 ± 0.08	**0.040**

Continuous variables are presented as mean ± standard deviation or median [interquartile range], whereas categorical variables are presented as absolute frequency (relative frequency). PWV: pulse wave velocity; BMI: Body Mass Index; HDL: High-density lipoprotein; LVDD: Left Ventricle Diastolic Diameter; LVSD: Left Ventricle Systolic Diameter; E: velocity of early diastolic mitral or tricuspid inflow; A: velocity of late diastolic mitral or tricuspid inflow; E’: mitral or tricuspid annular velocity of early diastole. CIMT: carotid intima-media thickness; Significance level: p<0.05.

## DISCUSSION

The great relevance of this study is its determination of **≥** 6.9 m/s as the cf-PWV cutoff value in PD patients, defined as a possible indicator of AS in this group. Definition of a subset with cf-PWV ≥ 6.9 enabled observation of positive relationships with blood glucose, obesity, dyslipidemia, and echocardiographic variables, such as early impaired LV diastolic function. Based on the results above, it can be speculated that cf-PWV may be an important vascular marker indicative of early signs of endothelial dysfunction in PD.

The cf-PWV measurement procedure is considered an important predictor of CVR, associated with AS. Studies show that endothelial dysfunction is triggered by hyperglycemia, which provokes increased levels of free radicals, proliferation of vascular smooth muscle cells, and increased advanced glycation end products (AGEs), culminating in a pro-inflammatory state and imbalance in the production of antioxidant substances, such as decreased NO synthesis. These changes lead to expression of metalloproteinase enzymes, which degrade elastin, increase generation of angiotensin-2 in vascular tissue and, consequently, favor greater arterial wall rigidity.^25,26^

Studies in the literature report cut-off values for the general populations of different nationalities. Many guidelines suggest different cf-PWV values according to individual CVR, such as cf-PWV > 10m/s for Europeans and cf-PWV > 18m/s for the Japanese,^27,28^ but none of these studies address a cf-PWV cutoff value for PD individuals. The values obtained in studies establish different PWV for evaluating CV changes. Markers that influenced the change in PWV were evaluated, such as the influence of dyslipidemia,^29^ which was also observed in this study with PD patients. Other studies demonstrate the importance of measuring arterial stiffness and adjusting blood glucose levels, through HbA1c values.^30^ Correlations with glycemia measured using HbA1c also confirmed changed PWV in DM patients.^31^ Other studies report PWV being used to assess risk markers and predictors of arterial stiffness, showing an early change in the elasticity of arteries when IR is involved.^32-34^ This was also observed in the present study. Another aspect also observed in the literature is obesity altering PWV in the general population,^35,36^ evaluating endothelial dysfunction in individuals divided into groups: control group without PD and without hypertension, another with only hypertensive patients, and one last group of patients with hypertension and PD. The last group showed greater changes in biomarkers that induce endothelial dysfunction and inflammation, with an increase in the levels of inflammatory molecules and cytokines, which are expressed with a slight increase in ICAM-1 and TNF-a. Although several studies in the literature presents hypertension in association with altered PWV,^37-39^ this was not observed in the present study with PD patients.

Wajchenberg^40^ showed that early discovery of endothelial dysfunction and early intervention can prevent long-term progression to coronary artery disease. Their work documents that the onset of endothelial dysfunction in patients with IR or with a high rate of developing DM2 was associated with hyperglycemia and increased oxidative stress, leading to formation of O_2_, which reacts with NO and leads to its degradation, favoring the process of increased peripheral vascular resistance and atherogenesis.

The limitations of this study were its small sample size and cross-sectional study design, with no follow-up of adverse outcome. Based on the limited sample, we can only suggest, as a pilot study, that PWV may have the potential to discriminate cardiovascular injury. The clinical implication is that early intervention in this population may make it possible to prevent cardiovascular complications.

## CONCLUSION

In PD patients, a cf-PWV cutoff value of 6.9 cm/s was discriminative for AS. These findings suggest that early investigation of cardiovascular injury and aggressive therapeutic strategy with good control of risk factors are worth pursuing in PD.
